# The Hippocratic Oath

**Published:** 1884-08

**Authors:** 


### The Hippocratic Oath.

"I swear by Apollo, the physician, by Aesculapius, by Hygeia and Panacea, and by all the gods and goddesses, that, to the best of my power and judgment, I will faithfully observe this oath and obligation. The master who has instructed me in the art I will esteem as my parents, and supply, as occasion may require with the comforts and necessaries of life. His children I will regard as my own brothers, and if they desire to learn I will instruct them in the same art without any reward or obligation. The precepts, the explanations, and whatever else belongs to the art I will communicate to my own children, to the children of my master, to such other pupils as have subscribed the physician's oath, and to no other persons. My patients shall be treated by me to the best of my power and judgment, in the most salutary manner, without any injury or violence; neither will I be prevailed upon by another to administer pernicious physic, or be the author of such advice, nor will I recommend to woman a pessary to produce abortion, but will live and practice chastely and religiously. Cutting for the stone I will not meddle with, but will leave it to the operators in that way. Whatever house I am sent for to, I will always make the patient's good my principal aim, avoiding as much as possible all voluntary injury and corruption, especially all venereal matters, whether among women or men, bond or free. And whatever I see or hear, in the course of a case or otherwise, relating to the private affairs of life, nobody shall ever know it if it ought to remain a secret. May I be prosperous in life and business, and forever honored and esteemed, by all men, as I observe and not confound this solemn oath; and may the reverse of all this be my portion if I violate it and forswear myself."

The above, though much criticised in these days, might in the main, with great propriety, be adopted and obeyed by the medical profession in general and special. If the dental profession should adopt and live up to it, it would round off and smooth up some of the sharp corners and rough places. How desirable that the principles here presented should guide any one who has to do with the health and life of his fellow man.—[Ed. Register.

